# Physiological, Biochemical, and Epigenetic Reaction of Maize (*Zea mays* L.) to Cultivation in Conditions of Varying Soil Salinity and Foliar Application of Silicon

**DOI:** 10.3390/ijms24021141

**Published:** 2023-01-06

**Authors:** Renata Tobiasz-Salach, Marzena Mazurek, Beata Jacek

**Affiliations:** 1 Department of Crop Production, University of Rzeszow, Zelwerowicza 4, 35-601 Rzeszow, Poland; 2 Department of Physiology and Plant Biotechnology, University of Rzeszow, Ćwiklińskiej 2, 35-601 Rzeszow, Poland

**Keywords:** carotenoids, chlorophyll fluorescence, gas exchange, methylation-sensitive amplified polymorphism (MSAP), stress

## Abstract

Soil salinity is one of the basic factors causing physiological, biochemical and epigenetic changes in plants. The negative effects of salt in the soil environment can be reduced by foliar application of silicon (Si). The study showed some positive effects of Si on maize plants (*Zea mays* L.) grown in various salinity conditions. At high soil salinity (300 and 400 mM NaCl), higher CCI content was demonstrated following the application of 0.2 and 0.3% Si. Chlorophyll fluorescence parameters (PI, F_V_/F_0_, F_v_/F_m_ and RC/ABS) were higher after spraying at 0.3 and 0.4% Si, and plant gas exchange (C_i_, P_N_, g_s_, E) was higher after spraying from 0.1 to 0.4% Si. Soil salinity determined by the level of chlorophyll *a* and *b*, and carotenoid pigments caused the accumulation of free proline in plant leaves. To detect changes in DNA methylation under salt stress and in combination with Si treatment of maize plants, the methylation-sensitive amplified polymorphism (MSAP) technique was used. The overall DNA methylation level within the 3′CCGG 5′ sequence varied among groups of plants differentially treated. Results obtained indicated alterations of DNA methylation in plants as a response to salt stress, and the effects of NaCl + Si were dose-dependent. These changes may suggest mechanisms for plant adaptation under salt stress.

## 1. Introduction

The reduction of agricultural arable land, growth in the human population, as well as climate change have necessitated a search for solutions that should increase plant productivity and resistance to environmental factors [1]. One of the possibilities for increasing plants yields up to 50% is improving the efficiency of photosynthesis [2]. However, this process is limited by various stress factors. Stress is most often caused by drought or soil salinity. These factors cause biochemical and physiological changes in plants, leading to disturbances in growth and development processes. The most common disorders of metabolic functions include photosynthetic inhibition, gas exchange, and growth inhibition. There are also changes in physicochemical cellular structures leading to the loss of the cell membrane’s ability in respect of selective permeability [3].

Numerous studies have indicated that any reduction in the chlorophyll content and carotenoids due to salinity stress can lead to decreases in the processes of photosynthetic efficiency [4,5,6]. Therefore, the analysis of chlorophyll *a*, *b*, total chlorophyll and carotenoids contents will enable the determination of the impacts caused by abiotic stress on plant growth in a relatively quick and simple way. Another type of stress detection may be the changes in free proline content, since there is increased synthesis of compatible substances, including proline [7,8,9], in plants grown under salt stress conditions. Thus, based on changes in proline content, it is possible to assess the physiological state and tolerance of plants under salinity stress. The damaging effect of salt in the soil also leads to the lowering of osmotic potential in soil solutions, which causes difficulties in the uptake of water and nutrients by plants. Several researchers [10,11] report that during salt stress, there are changes in the functioning of PSII, such as the lower ability to capture energy and the increased quenching of non-photochemical processes.

DNA methylation plays an important role in gene regulation during growth, development and different stresses. DNA methylation is an epigenetic modification in which the methylation of the cytosine base occurs during developmental and environmental responses by plants [12]. Changes in the methylation patterns of DNA during a cell’s lifetime provide an adaptive ability for the organism to adjust to changes in the environment [13,14]. Stress conditions lead to changes at morphological, physiological, biochemical as well as (epi)genetic levels [15]. The detection of methylation content during plant growth under biotic and abiotic stress has become popular and a valuable marker of plant response to stress. For maize plants, analyses of the level of DNA methylation in response to stress conditions were performed with respect to different stress conditions, such as: salinity [16,17]; herbicide treatment [18]; osmotic stress [17]; drought [19]; cold [20]; or ion treatment or deficiency [21].

DNA methylation is one of the earliest discovered and most studied regulatory mechanisms in epigenetics and is considered to be a relatively stable, heritable, transgenerational marker, involving a series of biological processes such as temporal and spatial gene expression, transposable element activity, and genomic imprinting [14,17]. The methylation-sensitive amplified polymorphism (MSAP) technique is widely used for researching the genome methylation status in response to stress [17,22,23]. MSAP is a modification of the amplified fragment length polymorphism (AFLP) technique in which the isoschizomers HpaII and MspI are used as restrictions enzymes that recognize the same restriction site (5′CCGG 3′), with different sensitivity to DNA methylation [24,25]. One of the methods to increase plant resistance to salt stress may be through foliar Si supply [1]. Si is the second most common element in the Earth’s crust after oxygen. It is a constituent of many rock-forming minerals [26]. The common forms of its occurrence are SiO_2_ and silicates [26]. Si has a positive effect on plant growth and is classified as a beneficial element [27,28,29]. Many plants from the *Poaceae*, *Equisetaceae* and *Cyperaceae* families accumulate more than 3% of Si in their tissues in relation to dry weight (Si-accumulator plants). High amounts of Si (up to 3% of dry weight) are also accumulated in cereals, including maize and wheat.

In recent years, considerable attention has been focused on the role of Si in increasing plant resistance to biotic and abiotic stress factors [15,30,31,32,33,34]. Some researchers consider Si to be a quasi-essential element [35].

Silicon affects the flexibility of cell walls and stiffness and protects against excessive water loss and the penetration of precipitates and pathogens in the detoxification of heavy metals. The positive effects of the presence of Si are demonstrable mainly under different stress conditions and when the Si concentration in plant tissues is high [28,36,37]. Supplying plants with soluble and easily assimilable Si compounds may, therefore, be one of the important methods in reducing the negative effects of environmental stress factors [1,29,35,38,39].

Maize is an important agricultural crop, coming third in global harvested area and global production [40]. It can be used for fodder, food, and industrial and energy purposes as well as in the phytoremediation process. This species is marked by relatively high water requirements and is a plant that is not very sensitive to salinity [33,41], but high accumulation of NaCl in the soil negatively affects its yield and growth [42]. It has been shown that foliar feeding of maize with Si leads to increasing water use efficiency by reducing leaf transpiration and the water flow rate in the xylem [43]. The use of Si in maize cultivation under stress influences the growth of leaf surfaces, delays their aging process and improves grain quality [41,42,43,44]. Due to the many benefits of Si in plants (especially under stress conditions), analysis of this element should be enhanced. Therefore, the current research was carried out to determine the effect of Si foliar application in different concentrations on the activity of the photosynthetic apparatus, gas exchange, proline content and the DNA methylation level in maize cultivated in varied salinity conditions.

The research hypothesis assumed that foliar application of Si in maize cultivation under salinity conditions will partially reduce the negative effects of salt on physiological parameters, plant gas exchange, and free proline content, and hence, lead to changes in DNA methylation levels.

## 2. Results

### 2.1. The Effect of Foliar Application of Silicon on the Relative Chlorophyll Content in Maize Leaves Cultivated in Varying Soil Salinity Conditions

**Figure 1:**
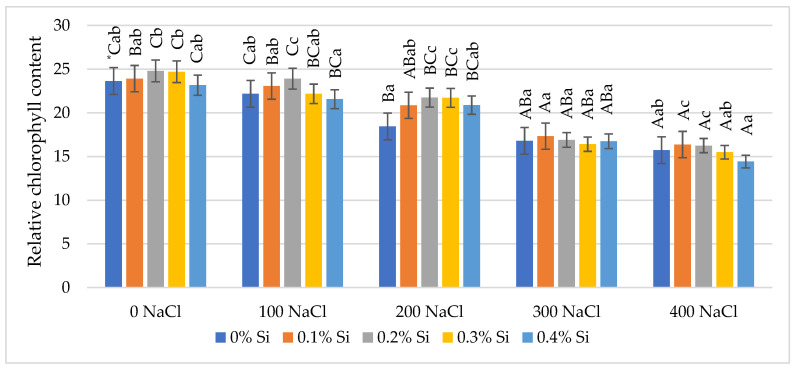
The effect of Si on relative chlorophyll content in maize leaves grown under varied salinity levels. * Capital letters represent significant differences between the averages for soil salinity at the same doses of Si; lowercase letters represent the mean significant differences between the doses of Si at the same salinity (*p* = 0.05).

The relative chlorophyll content (CCI) in leaves depended on salinity conditions and silicon foliar application. The analysis of variance showed a negative effect of salt on the chlorophyll content in maize leaves. It has been proven that decreasing CCI was dependent on increasing salinity content. The CCI index decrease is shown in Figure 1. A significant reduction of CCI in comparison to the control at the highest salt doses (300 mM NaCl and 400 mM NaCl) was observed. Foliar application of Si resulted in increased chlorophyll content, which was, however, dependent on the soil salinity degree. The most dominant effect of Si on the content of chlorophyll under stress conditions was demonstrated at 100 mM NaCl and 200 mM NaCl salinity levels. At a salinity of 100 mM NaCl, a significant increase in the CCI value (by 7.2%) was demonstrated after application of 0.2% Si, compared to plants without Si applied. However, at a salinity of 200 mM NaCl, the increase was observed after spraying 0.2% and 0.3% Si. At the highest soil salinity (400 mM NaCl), an increase (mean 4.5%) in CCI was also demonstrated with foliar application of 0.1% Si and 0.2% Si (Figure 1). This dependence proved the positive effect of Si on the accumulation of CCI in maize leaves growing under varying salinity conditions.

### 2.2. Effect of Foliar Application of Silicon on Chlorophyll Fluorescence Parameters in Maize Leaves Cultivated under Varying Salinity Conditions

**Figure 2:**
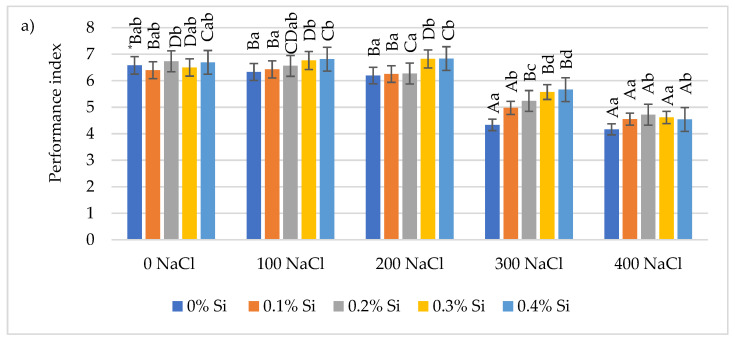

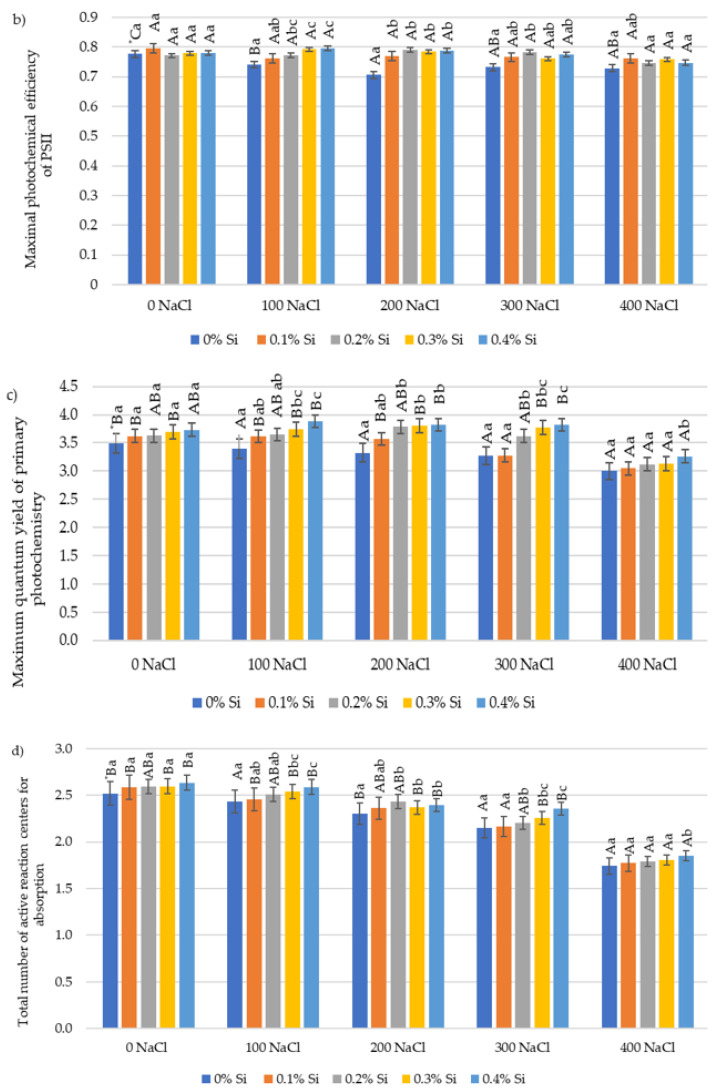
The effect of Si on chlorophyll fluorescence parameters: PS II performance index (**a**), maximal quantum yield of PSII photochemistry (**b**), maximum primary photochemistry yield (**c**) and total number of active reaction centers for absorption (**d**) in maize leaves grown under salinity stress. * Capital letters represent significant differences between the averages for soil salinity at the same doses of Si; lowercase letters represent the mean significant differences between the doses of Si at the same salinity (*p* = 0.05).

The performance index (PI) value depended on soil salinity and was modified by Si foliar application. The analysis of variance showed a negative effect of salinity on this indicator. A significant decrease in PI by an average of 21.7% and 31.3%, respectively, compared to the control (without NaCl) was shown at higher doses of salt (300 and 400 mM NaCl). The foliar application of Si limited the negative impact of salt on this parameter in each salinity variant. At lowest (100 mM NaCl) and moderate (200 mM NaCl) salinity conditions, the application of Si (0.3% and 0.4% Si dose) significantly increased the PI value (up 6.8% and 9.2%, respectively) compared to the control. In the 300 mM NaCl variant, a significant increase in PI was demonstrated after each foliar application of Si doses. A significant increase was observed with the application of Si 0.2% (10.8%) and 0.4% Si (8.2%) (Figure 2a) in conditions of highest salinity (400 mM NaCl).

The analysis of variance for the chlorophyll fluorescence index (F_v_/F_m_) showed a decrease (3.9 and 9.1%, respectively) in this parameter compared to the control, under 100 and 200 mM NaCl soil salinity conditions. Foliar application of Si caused an increase in the F_v_/F_m_ index using Si in doses of 0.2, 0.3, and 0.4%, with lower and moderate soil salinity (100 and 200 mM NaCl). The positive effect of Si was also observed at higher salinity (300 and 400 mM NaCl) levels, but this increase was not statistically significant (Figure 2b).

Experimental factors also affected the F_v_/F_0_ parameter. In the case of plants growing under salinity conditions (without Si), lower values of the measured parameter were noted compared to the control. This relationship was especially visible at the highest salinity (400 mM NaCl) level. Foliar Si application in each salinity variant resulted in an increase in F_v_/F_0_ values. The most significant rise was observed from the 0.3 and 0.4% Si dose applications (Figure 2c).

Soil salinity had a negative effect on the RC/ABS value. For the plants growing in high salinity conditions (variant 300 and 400 mM NaCl), a significant decrease in the RC/ABS value in comparison to the control (without NaCl) was observed. A slight decrease in this parameter was also obtained at lower salt concentrations, but it was statistically non-significant. Analyzing the effect of foliar application of Si, its positive effect was observed at the lowest salinity (100 mM NaCl—using 0.2 and 0.3% Si sprays) and the highest (400 mM NaCl—0.2% Si). In the case of other variants of the experiment, no significant impact on RC/ABS was confirmed.

### 2.3. Effect of Foliar Application of Silicon on the Gas Exchange Parameters of Maize Leaves Cultivated under Varying Salinity Conditions

**Figure 3:**
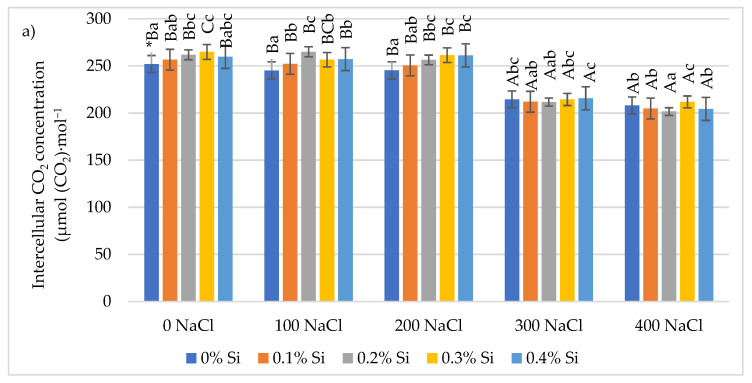

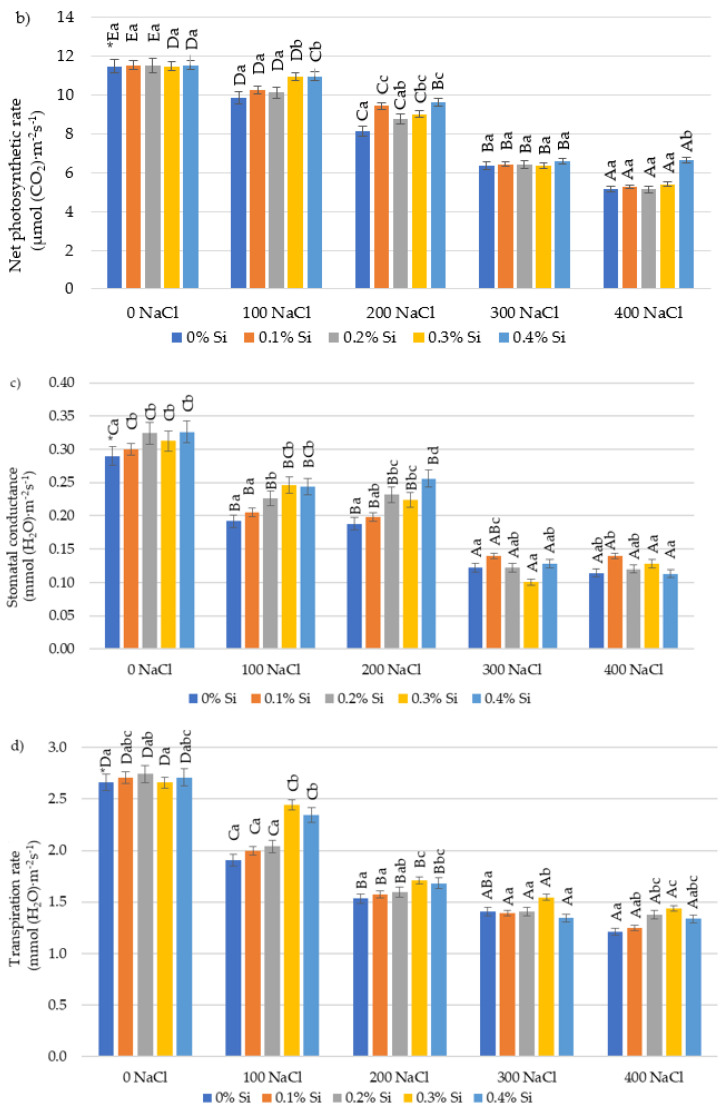
The effect of Si on gas exchange parameters: (**a**), intercellular CO_2_ concentration (**b**), transpiration rate (**c**) stomatal conductance (**d**) net photosynthetic rate in maize leaves cultivated in varying salinity conditions. * Capital letters indicate significant differences between the mean values for soil salinity at the same doses of Si. The lowercase letters represent the mean significant differences among the doses of Si at the same salinity (*p* = 0.05).

The analysis of variance showed the influence of soil salinity and foliar application of Si on the C_i_ parameter values. The lowest C_i_ values were obtained for plants growing under soil salinity conditions of 300–400 mM NaCl levels. Using NaCl, at 400 mM NaCl, the reaction of plants to foliar application of Si was different. Si at 0.2% and 0.3% doses led to a 3.6% decrease and 1.8% increase in C_i_, respectively, following the application of Si. It was statistically proven. With lower (100 and 200 mM NaCl) salinity conditions, an increase in the C_i_ parameter was observed after each dose of Si foliar application (Figure 3a). Soil salinity had a negative impact on the P_N_ index, causing its decrease. Decreases were noted for plants grown on different variants of salinity. The highest values were obtained under the highest (400 mM NaCl) salinity conditions. Foliar application of Si had a positive effect P_N_ value increases. It was mostly observed at lower salinity levels. At 100 mM NaCl salinity conditions, the beneficial effect of Si was noted with the application of Si at 0.3 and 0.4% levels. P_N_ increases under 200 mM NaCl soil salinity were due to foliar application of Si. The highest increase (22.4%) was demonstrated with foliar application of Si at the 0.2% level. The use of Si in the conditions of high soil salinity (variant 300 and 400 mM NaCl) did not cause changes in the value of the P_N_ index (Figure 3b).

Stomatal conductance (g_s_) was determined by the experimental factors (NaCl and Si) used. Higher soil salinity resulted in a decrease in the g_s_ parameter value in comparison to the control (without NaCl). The lowest values of the g_s_ parameter were obtained at the highest soil salinity (300 and 400 mM NaCl) levels (Figure 3c). Foliar application of Si caused an increase in the measured parameter in the control (without salt) as well as in situations with soil salinity. The highest g_s_ values were obtained at Si—0.1%; 0.3% and 0.4% (at 100 mM NaCl salinity) and 0.4% Si (at 200 mM NaCl salinity). No effect of Si on the g_s_ parameter values was demonstrated in the higher soil salinity conditions (variant 300 and 400 mM NaCl) (Figure 3c).

The transpiration rate (E) parameter for maize plants grown under salinity conditions was lower than in the control (without NaCl). Soil salinity at the levels of 300 and 400 mM NaCl caused a significant decrease in E values, in comparison to the control and with lower salt concentrations (100 and 200 mM NaCl) (Figure 3d). Under lower soil salinity conditions (100 and 200 mM NaCl), the use of higher concentrations (0.3 and 0.4%) of Si resulted in increased E values. A positive effect of Si on the E index was observed after applying 0.3% Si in higher soil salinity (300 and 400 mM NaCl) conditions (Figure 3d).

### 2.4. Effect of Foliar Application of Silicon on the Content of Chlorophyll a and b and Carotenoids in Maize Leaves Cultivated in Conditions of Varied Salinity

**Figure 4:**
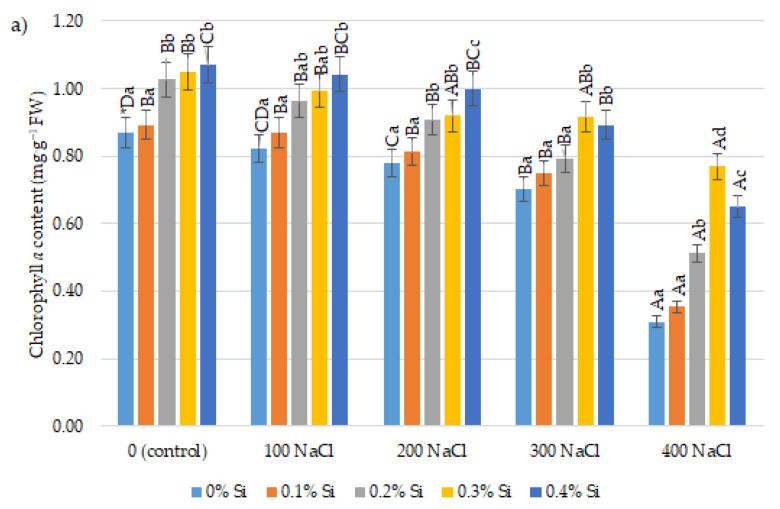

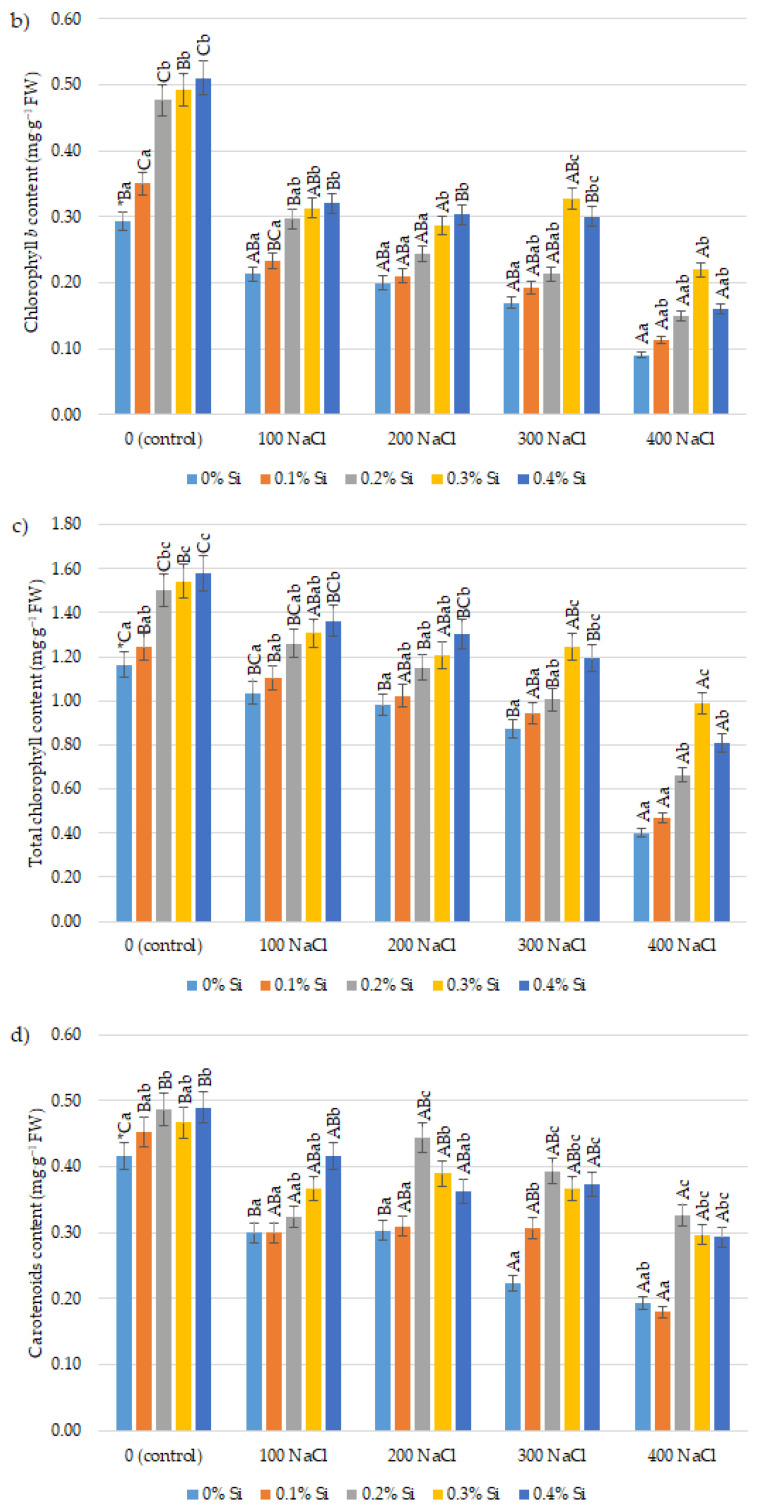
The effect of Si on the chlorophyll *a* (**a**), *b* (**b**), total (**c**) and carotenoids (**d**) content in maize leaves cultivated in varying salinity conditions. * Capital letters indicate significant differences between the means for soil salinity at the same doses of Si, and lowercase letters represent the mean significant differences between the doses of Si at the same salinity (*p* = 0.05).

Chlorophyll *a* content in maize leaves was reduced under salt stress. The significantly lowest values were obtained at 400 mM NaCl (Figure 4a). The content of chlorophyll *a* was almost three-fold lower at the highest doses of salinity without foliar Si application in comparison with the control plants. The foliar application of Si generally increased the chlorophyll *a* content in maize plants. The use of Si at a concentration of 0.3% at 400 mM NaCl caused a significant increase in chlorophyll *a* content in comparison with control plants without Si application. At the highest soil salinity of 400 mM NaCl, increases in this parameter were also found with foliar application of 0.2 and 0.4% Si (Figure 4a).

Maize plants grown under salinity conditions (without the Si addition) were characterized by a lower chlorophyll *b* content in comparison with the control plants. This was especially visible at the highest salinity (400 mM NaCl) level. At lower salt concentrations (100–300 mM NaCl), lower values of this parameter were also recorded compared to the control (without NaCl treatment). Nonetheless, the foliar application of Si generally increased the content of chlorophyll *b* (Figure 4b). The application of Si at 0.3% at 400 mM NaCl concentration caused a significant increase in chlorophyll *b* in comparison to the control. The positive effect of 0.3 and 0.4% Si doses in comparison to the control, without Si application, was also found in the lower soil salinity conditions (100–300 mM NaCl).

Total chlorophyll content significantly decreased in response to the application of 400 mM NaCl compared to the no-salt variant. Plants grown in these salinity conditions were characterized by almost three-fold lower chlorophyll *a* and *b* content in comparison with control plants. The foliar application of Si limited the negative effect of the salt in each experimental variant (Figure 4c). Significantly higher values of chlorophyll *a* + *b* were obtained after the application of 0.3% Si in comparison to the control plants (without Si) at 400 mM NaCl. In such salinity conditions, significantly higher values were also found at the concentrations of 0.2 and 0.4% Si in comparison with no Si application and the smallest dose of 0.1% Si. The positive effect of silicon on total chlorophyll content was also observed at lower salinity (100–300 mM NaCl) levels.

Carotenoids content in maize leaves depended on the salinity conditions and silicon dose applications (Figure 4d). The significantly lowest parameter values were obtained at 400 mM NaCl in comparison with control plants (without Si application). However, the application of Si in the concentration of 0.2% at 400 mM NaCl caused a significant increase in carotenoids content in comparison to the 0.0% Si variant. Likewise, use of 0.2 and 0.3% Si at 200 and 300 mM NaCl had an impact on the increase in carotenoids content as well.

### 2.5. The Effect of Foliar Application of Silicon on Proline Content in Maize Leaves Grown under Varied Salinity Conditions

**Figure 5:**
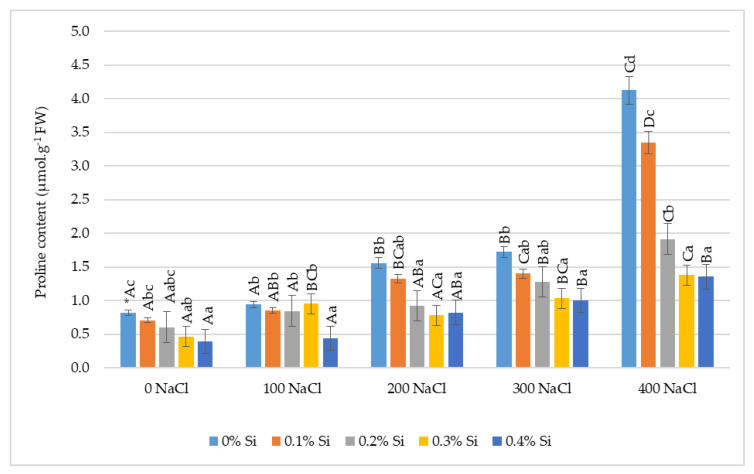
The effect of Si on the free proline content in maize leaves cultivated in varying salinity conditions. * Capital letters indicate significant differences between the means for soil salinity at the same doses of Si, and lowercase letters represent the mean significant differences between the doses of Si at the same salinity (*p* = 0.05).

The content of free proline in leaves depended on salinity conditions and silicon dose. The values of this parameter were increased under salt stress conditions. The significantly highest values were obtained at the highest salt dose (Figure 5). The free proline content was five-fold higher at 400 mM NaCl without Si application in comparison with the control plants. At the salinity levels of 200 and 300 mM NaCl (without Si), a significantly higher concentration of this amino acid was also found compared to the control. Silicon foliar application significantly reduced the content of free proline in maize leaves at each NaCl concentration in comparison to plants without fertilization. The use of Si at a dose of 0.4% resulted in significantly lower proline accumulations in the leaves in comparison with control plants without Si, despite their cultivation in similar salinity conditions.

### 2.6. Effect of Foliar Application of Silicon on DNA Methylation in Maize Plants Cultivated in Conditions of Varied Salinity

**Figure 6:**
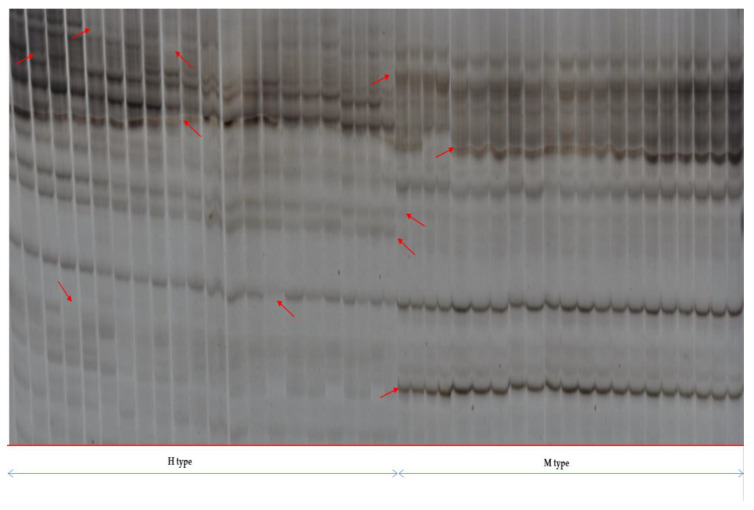
Comparison of representative MSAP bands (products of selective amplification of the EcoRI-AT × MspI/HpaII-ATG primer combination used). H and M refer to digestion with EcoRI + HpaII and EcoRI + MspI, respectively. Red arrows show polymorphic bands.

MSAP analyses were performed using five combinations of selective EcoR I and Msp I/Hpa II primers. An electropherogram photo showing the results of DNA methylation profiles of maize is presented in Figure 6. As can be seen, a highly repetitive banding pattern of the amplification products can be observed. There were also detectable changes in DNA methylation profiles between samples derived from maize cultivated in varied ranges of salinity (100–400 mM NaCl) in combination with Si (0.1–0.4% Si). Differential bands were attributed to the changes in DNA methylation between maize samples. The methylation level in the case of the control condition was 53%. Applications of Si in the control conditions led to slight increases in levels of total methylation in the range of 55–59.6%. However, in the case of 0.3% Si, the total content of methylation was comparable with that of the control. In salinity stress conditions, methylation frequency varied depending on the NaCl and Si doses. The highest values of total methylation (62.1–64.4%) were obtained in the condition of high salinity (400 mM NaCl). On the other hand, the lowest values (41.8–45.7%) were obtained for the 100 mM NaCl dose. Moderate salinity conditions (200–300 mM NaCl) were characterized by an intermediate frequency of DNA methylation (50–59.6%).

The numbers of hemi-methylated and fully methylated cytosines at 5′CCGG′3 restriction sites were also calculated. In almost every analyzed sample, the predominant tendency of hemi-methylated events was observed (Table 1).

Total percentage of DNA methylation was calculated and presented in Table 1.

## 3. Discussion

Soil salinity is one of the main factors causing abiotic stresses in plants. It negatively affects photosynthesis and the biochemical and epigenetic processes of plants. Numerous studies indicate that salt causes changes in the overall efficiency of photosynthesis [45], which was also shown in the current research. In comparison with the control (without salt), changes in photosynthesis parameters such as chlorophyll fluorescence and plant gas exchange were observed in all of the salinity variants. High levels of salt cause cell organelle damage and changes in cellular metabolism [46,47,48]. Na^+^ and Cl^−^ ions increase the accumulation of chemical compounds such as 0_2_ (singlet oxygen) or H_2_O_2_ (hydrogen peroxide) in plant cells. These compounds cause oxidative stress. Nucleic acids, proteins and lipids are damaged as an effect of oxidative stress [49,50]. In addition, when plants are exposed to salinity stress, energy requirements can rise significantly to run several energy-intensive adaptive mechanisms ensuring ion homeostasis, osmotic regulation and ROS defense [51]. Exaggerated dissipation of photosynthetic energy may play a huge role in defensive photoinhibition, photodamage, and photooxidative salinity tolerance [52]. The response of plants to soil salinity differs and depends on the species and salinity status of the soil [53]. Maize is a species resistant to salinity, but a high accumulation of NaCl in the soil negatively affects its yield and growth [42].

The results of the undertaken research indicate the negative impact of soil salinity on relative chlorophyll content in leaves (CCI). The chlorophyll content was lower in plants growing under high concentrations of NaCl (300 and 400 mM NaCl) in comparison to the control. Foliar application of Si caused increases in the chlorophyll content, although this depended on the soil salinity degree.

The most visible effect of Si application on the relative chlorophyll content in leaves was demonstrated at 100, 200, and 400 mM NaCl concentrations. In the lower soil salinity (100 and 200 NaCl), a significant increase was observed after spraying with 0.2 and 0.3% Si doses, in comparison with plants without Si sprayed. Furthermore, in the highest soil salinity condition (400 mM NaCl), such an increase in CCI was demonstrated after spraying with 0.1 and 0.2% Si.

The results of our research are similar to those presented by other researchers [42,54,55,56,57,58,59]. The reduction in photosynthetic pigments can be caused by the breakdown of the thylakoid membrane through the formation of proteolytic enzymes. The mentioned enzymes are responsible for the degradation of chlorophyll, as well as damage to the photosynthetic apparatus [45,60].

Chlorophyll content decreased after salt treatment. However, the application of Si resulted in an increase in chlorophyll content, which was also demonstrated by other researchers, including Kalteh et al. [61] and Chung et al. [62].

The method of assessing metabolic dysfunction and structural changes in plants is the measurement of chlorophyll fluorescence, which registers fluorescence *a* (reemission of light energy absorbed by the energy antennas of the photosynthetic apparatus). Fluorescence measures permit the determination of photosynthetic activity as well as changes in the general bioenergetic conditions of photosynthetic organisms under abiotic stress conditions [9,11,63,64]. The research conducted, especially with higher variants of soil salinity, indicated decreases in the chlorophyll fluorescence parameters such as PI, F_v_/F_0_ and RC/ABS. The negative impact of salt was restricted by foliar application of Si. Analyses performed by Kumar et al. [65] and Souri et al. [66] indicate that Si in the powdered form easily penetrates into the cell through the stomata, entering into biochemical reactions and improving its condition. Similar results were observed in our analysis.

NaCl stress can limit the efficiency of PSI and PSII and disrupt the biochemistry of photosynthesis, due to impaired chloroplast integrity [67,68]. It was also proved in our research by observing declines in chlorophyll content. This phenomenon was reported also by Zeng et al. [54], Zeeshan et al. [58] and Xia et al. [69].

The results of the performed analysis and undertaken literature research indicated that increased soil salinity causes many changes in energy processes, inactivates reaction centers, and inhibits electron transport, while foliar application of Si limits the negative effects of salt. Subsequently, it will be recommended to treat plants with Si several times during the growing season [15,70].

In the current research, parameters such as gas exchange, significant decreases in C_i_, P_N_, g_s_, and E assimilation by plants under salt stress may be caused by limited CO_2_ supply, which is due to the partial closure of the stomata and an impaired biochemical process of CO_2_ fixation [71]. To avoid excessive water loss, plants respond to salt stress by reducing stomatal conductance [72]. This situation causes photosynthetic CO_2_ fixation to be reduced, leading to a change in cellular metabolism and a rise in ROS production in the chloroplasts. ROS can lead to the damage of the photosynthetic apparatus, especially PSII, causing photoinhibition due to an imbalance in photosynthetic redox signaling pathways and inhibition of PSII repair [72,73,74,75]. In the current research, the loss in C_i_ caused by the increase in soil salinity most likely caused an exemption to the Calvin cycle reaction and induced photorespiration. There was a reduction in oxidized NADP+, which performs the role of a terminal electron acceptor in PSI and alternatively increases O_2_ electron leakage to form O_−2_, resulting in more H_2_O_2_ formation in the peroxisome. Similar dependences were shown by other researchers [76,77]. Stadnik et al. [15] and Zeeshan et al. [58], for example, observed reductions in stomatal conductance (g_s_) and photosynthesis rate (P_N_) in barley plants under salinity conditions.

The decrease in g_s_, P_N_ and E under the influence of salinity was proved by other researchers [58,78,79], and salt tolerance was controlled at the cellular level by manipulating the accumulation of P_N_, E, and g_s_ along with increased production of antioxidant enzymes (POX and SOD). In the current research, the application of Si, especially at higher doses, led to increases in the measured parameters. This phenomenon was confirmed by Yeo et al. [80], Li et al. [81] and Chung et al. [62]. Si led to the induction and rise in g_s_ and P_N_, due to superior ultrastructural organization of chloroplasts. The beneficial effect of Si application on the photosynthetic apparatus and the increased activity of photosynthesis can be linked with plants’ greater K^+^ uptake capacity and enhanced antioxidant defense [82]. Foliar application of Si on sorghum plants led to an increase in gas exchange parameters. Similar outcomes were also observed by de Oliveira et al. [83]. Rios et al. [84] posit that Si improves root hydraulic conductivity and stomatal function by regulating aquaporins.

In order to analyze, more precisely, the influence of Si on the physiological state of plants cultivated in salinity conditions, real chlorophyll and carotenoids were determined. In our research, the salinity caused a decrease in chlorophyll *a* and *b* and total chlorophyll content in maize plants. The significantly lowest parameters were found at high soil salinity, which was confirmed in the available literature. Aliu et al. [85] recorded significantly lower content of chlorophyll *a, b* and *a + b* for maize plants grown at 400 mM NaCl. However, three lower doses of NaCl led to increases in the content of these assimilated pigments in comparison with control plants. Decreased values of these parameters at high salinity (300 mM) in maize plants were confirmed by Di et al. [86]. Similarly, in the research performed by Raza et al. [87], chlorophyll *a* and *b* content significantly decreased in salt stress conditions, compared with control plants. In our experiment, foliar application of Si increased chlorophyll *a*, *b,* and total chlorophyll as well as carotenoid content in the leaves of maize plants cultivated in varied soil salinities in comparison with the control (without Si). As a result, it can be inferred that the application of Si reduces the negative effect of high soil salinity on plant growth. Similarly, in the studies presented by Xie et al. [33] and Barbosa et al. [88], the application of Si increased the content of chlorophyll. Increased content of chlorophyll in maize plants grown under salinity conditions and treated with Si simultaneously was also noted by Rohanipoor et al. [89] and Khan et al. [68].

One of the reactions of plants to salinity stress is the observed increase in the accumulation of compatible substances, including proline that supports plants’ adaptation to stress conditions. Many studies [78,90,91] indicate that proline accumulation occurs in plants under salt stress. In our research, free proline content was five-fold higher at 400 mM NaCl without Si application in comparison with the control plants. Similarly, in the study by Cha-Um et al. [90], the highest content of this amino acid was found at 400 mM NaCl, and it was almost four-fold higher than in the control. A significant increase in the proline content in maize plants grown under salinity stress was also reported by Carpici et al. [92], Agami [93] as well as Molazem and Bashirzadeh [94]. Similarly, a significant increase in free proline content in barley under soil salinity was found [7]. Available data in the current literature support the argument that foliar application of Si reduces the negative effects of high salinity. Our research shows that the application of Si at a dose of 0.4% caused a significant decrease in proline content, which may indicate an increase in plants’ tolerance to salinity. A decrease in this parameter for plants that were grown under salinity conditions after the application of Si was also noted by Natarajan et al. [95] and Moussa [96]. Similarly, in the studies by Delavar et al. [97] and Parveen et al. [98], the addition of Si decreased the content of free proline in maize plants.

Epigenetic changes in plants are suspected to be a mechanism for the initiation and regulation of their potential defense metabolism [99]. More and more scientific reports indicate a significant role of epigenetic changes under stress conditions [17,22,23,100,101]. Epigenetic variation describes changes that are not attributable to changes in DNA sequence. Cytosine methylation is one of the molecular mechanisms that can contribute to epigenetic variation and often acts to suppress the activity of active genes, transposable elements, repetitive sequences, and pseudogenes [101,102]. In the current research, we obtained a methylation frequency level of 53.4% in control conditions. Data from the available literature showed similar levels of DNA methylation in maize plants in stress-free conditions, with genotype dependency. According to Tyczewska [40], the total methylation level of 5-CCGG-3 sequences of maize under the control conditions averaged between 63.11% intolerant and 59.38% in lines sensitive to herbicidal stress. Research performed by Eichten et al. [102] using methylated DNA immunoprecipitation techniques enabled the detection of methylation levels in two maize genotypes (B73 and Mo17), at 50.11% and 41.37%, respectively.

Several environmental and genetic stimuli are known to alter methylation. Abiotic stress can cause increases or decreases in cytosine methylation throughout the genome and at specific loci [103].

It was reported that in maize under salinity stress, thousands of genes involved in cellular processes, metabolic processes, and signal transduction were associated with differential DNA methylation [16]. Research performed by Mager and Ludewig [104] indicated that an immense loss of DNA methylation was reported under nitrogen or phosphate deficiency during growth of maize plants.

According to Eichten et al. [102], methylation patterns within maize gene bodies are similar to the density of CG sites. Research performed by Tyczewska et al. [18] that focused on analyzing DNA isolated from herbicide-treated maize plants revealed that changes in DNA methylation profiles occurred in genes encoding heat shock proteins, membrane proteins, transporters, kinases, lipases, methyltransferases, zinc-finger proteins, cytochromes, and transposons. Furthermore, Tyczewska et al. [18] indicated that large increases in DNA methylation levels in the sensitive line resulted in a lower ability to cope with stress conditions.

This seems to corroborate the results obtained in the current research indicating increases in methylation frequency along with increasing salinity. The relationship between DNA methylation and gene expression was also analyzed by other researchers [102,105]. For example, Li et al. [106] postulated that highly expressed genes during leaf development in maize are an indication of very low levels of methylation.

Declining methylation frequency obtained for maize plants under salinity conditions induced by 100–300 mM NaCl, in combination with varied doses of Si (Table 1), may indicate the activation of genes responsible for coping with stress conditions.

This phenomenon was not observed under the high salinity condition (400 mM NaCl) in which total methylation was observed to have increased (Table 1).

According to research [49,74,107], pre-exposing plants to various abiotic stresses, i.e., high salt, mild or high temperature, cold, or water withdrawal, may cause altered responses to future stresses. It is determined as a plant’s epigenetic memory. The epigenetic memory of a cell defines the set of modifications in the cell’s DNA, but does not alter the DNA sequence. These modifications can alter gene expression, and hence the characteristics and genetic behavior of the cell. Ding et al. [107] proved that maize and *Arabidopsis thaliana* L. that had been subjected to several dehydration/rehydration cycles displayed improved retention of water compared to plants experiencing the first stress. According to the above, it can be assumed that maize plants subjected to moderate salinity stress in combination with Si improve the response to stress conditions in the next round of salinity. Along with transgenerational epigenetic inheritance, these changes can be passed down to an organism’s offspring. So, it is not the sequence that could be changed, but its functioning [100,108].

## 4. Materials and Methods

### 4.1. Plant Growth Conditions

The pot experiment in controlled conditions was carried out at the Department of Plant Production at the University of Rzeszów (Poland). Eight maize seeds were sown in plastic pots with a diameter of 25 cm. Six kilograms of soil with a clay-sand grain size and a slightly acidic reaction (KCl pH 6.31; H_2_O 6.50) were placed in the pots. The total contents of compounds in the soil were: 16.4 mg·100 g^−1^ P_2_O_5_, 16.5 mg·100 g^−1^ K_2_O, 8.81 mg·100 g^−1^ Mg, and 9.51 mg·100 g^−1^ Ca. After emergence, the number of maize plants per pot was five. The experiment was carried out in a growth chamber (Model GC-300/1000, JEIO Tech Co., Ltd., Seoul, South Korea) at a temperature of 21 ± 2 °C, relative humidity of 60 ± 3%, and a photoperiod of 16:8 h of light/darkness. The two-factor experiment was set up in a completely randomized design (CRD) with four replications. Pot positions in the experiment were randomized every week.

An aqueous solution of NaCl with the concentrations of 100, 200, 300, and 400 mM was applied to the soil (after plants’ emergence (two-leaf stage)) in a volume of 50 cm^3^ per pot. Plants without salt addition and without Si foliar spray were treated as controls. After 12 days from the application of the NaCl solution to the soil, silicon (Optysil content—200 g L^−1^ SiO_2_) was applied to the leaves. The spray was applied in four concentrations: 0.1, 0.2, 0.3, and 0.4% Si. The spraying was carried out with a manual laboratory sprayer with an adjustable stream with a dose of 1.2 mL ± 0.1 during one press (outlet diameter 0.6 mm). A uniform spraying procedure was used, and the plants were sprayed until the measured dose was completely utilized. In the control sample, deionized water was used at the same time. Physiological measurements (relative chlorophyll content, chlorophyll fluorescence, and gas exchange) on maize plants were taken at the first or second fully developed leaf stage, 2 and 4 days after the foliar application of Si.

### 4.2. Measurement of Physiological Parameters

#### 4.2.1. Relative Chlorophyll Content

Measurements were made using a hand-held CCM-200plus Chlorophyll Meter (Opti-Sciences, Hudson, NH, USA). They were made on fully exposed maize leaves. Five leaves per pot were analyzed (50 measurements per concentration).

#### 4.2.2. Chlorophyll Fluorescence

Measurements of chlorophyll fluorescence in leaves were taken using a fluorimeter Pocket PEA (Pocket PEA, Hansatech Instruments, King’s Lynn, Norfolk, UK). Dark adaptation clips were applied to the leaf blade (except the leaf nerve) for 30 min. The following parameters were measured: maximum quantum yield of PSII photochemistry (F_v_/F_m_), maximum quantum yield of primary photochemistry (F_v_/F_0_), photosynthetic efficiency index (PI), and total number of active reaction centers for absorption (RC/ABS). The fluorescence signal was collected in actinic red light with a peak light source wavelength of 627 nm and transmitted for 1 s at the maximum available intensity of 3500 μmol (photon) for photosynthetically active radiation (PAR) m^−2^ s^−1^ [109,110]. Fluorescence measurements were performed in each pot on five leaves with four replicates.

#### 4.2.3. Gas Exchange

The LCpro-SD photosynthesis measurement system (ADC Bioscientific Ltd., Herts, UK) was used to measure the photosynthesis of the leaves. The LCpro-SD Plant Leaf Photosynthesis Chamber has a flow accuracy of ±2% of its range. During the measurement, the light intensity in the measuring chamber was 350 µmol m^−2^·s^−1^, and the temperature was 23 ± 2 °C. Net photosynthesis rate (P_N_), transpiration (E), stomatal conductance (g_s_) and intercellular CO_2_ concentration (C_i_) were analyzed on five fully exposed leaves in four replicates.

### 4.3. Determination of the Chlorophyll, Carotenoids and Free Proline Content

The real chlorophyll and carotenoid content in the fresh leaf mass was determined by the spectrophotometric method according to Hiscox and Israelstam [111]. For this purpose, a weighed sample of leaf mass (~100 mg) was extracted with 5 cm^3^ of dimethyl sulfoxide (DMSO) and then incubated for 30 min at 65 °C. In the obtained extract, the content of chlorophyll *a, b* and total chlorophyll as well as the carotenoids was determined using the AquaMate Vis spectrophotometer at 470, 645 and 663 nm wavelengths. Chlorophyll content was calculated following the equations used by Arnon [112], while the carotenoid content was determined based on the equation proposed by Lichtenthaler’a [113].

The free proline concentration was determined in the reaction of proline with acid ninhydrin according to the method described by Bates et al. [114]. The weighed sample of leaf mass (~0.5 g) was homogenized with 10 cm^3^ of 3% sulfosalicylic acid. A 2 cm^3^ measure of the obtained supernatant was reacted with 2 cm^3^ of glacial acetic acid and 2 cm^3^ of ninhydrin reagent for 1 h at 100 °C. After the incubation, the samples were placed on ice for 5 min. Then, 4 cm^3^ of toluene was added to the extract and shaken for about 20 s. After the layers were separated, the absorbance was measured at 520 nm using toluene as a blank, and the free proline concentration was calculated from a standard curve prepared from a series of proline standard solutions.

### 4.4. MSAP Electrophoresis and Visualization and Methylation Analysis

The plant materials used in this research included fully developed leaves of maize, collected on the last day of the experiment. Genomic DNA was extracted using the hexadecyltrimethylammonium bromide (CTAB) protocol [115] with some slight modifications. The MSAP analysis was performed using the protocol described by Peraza-Echeverria et al. [24] and Xiong et al. [25] with some modifications. The isoschizomers HpaII and MspI enzymes were used to detect cytosine methylation. These isoschizomers recognize the sequence 5′CCGG 3′. The capacity to cleave at the recognized sequence is dependent on the methylation state of the external or internal cytosine residues. HpaII is inactive if one or both cytosines are fully methylated (both strands methylated; symmetric methylation) but cleaves the hemi-methylated sequence (only one DNA strand methylated), whereas MspI cleaves 5′C^m^CGG 3′ but not 5′^m^CCGG 3′ [24,25]. At the beginning, two independent digestion reactions for each genomic DNA sample were carried out. In the first reaction, 0.5 µg of the genomic DNA was digested with 10 U of EcoRI (Thermo Scientific, Waltham, MA, USA) plus 10 U of MspI (Thermo Scientific) and 1× Tango buffer (Thermo Scientific) in a final volume of 20 µL for 6 h at 37 °C. The second digestion reaction was carried out as above; however, HpaII (Thermo Scientific) was used instead of MspI.

The products of digestion were then ligated to the adapters by adding 30 µL of ligation mixture containing 1U T4 DNA ligase (Invitrogen), 1 × T4 DNA ligase buffer (Invitrogen), 10 pmol EcoRI adapter (Genomed) and 50 pmol MspI-Hpa II adapter (Genomed) (Table 2). The ligation reaction was performed at 20 °C overnight.

A 2.5 µL amount of the obtained ligation product was used for the preamplification reaction, in combination with 0.5 µM Pre-MspI-HpaII primer (Genomed) and 0.5 µM Pre-EcoRI primer (Genomed), 200 µM each of dNTP, 1× PCR buffer (Dream Taq, Thermo Scientific) and 1 U Taq polymerase (Dream Taq, Thermo Scientific), in a final volume of 20 µL. The reaction consisted of: denaturation at 94 °C for 30 s, primer annealing at 46 °C for 1 min, primer extension at 72 °C for 1 min, and final extension at 72 °C for 5 min. Extensions of the DNA strands from primers were carried out for 30 cycles of PCR.

Selective amplification was conducted in volumes of 20 µL. The selective amplification reaction was performed by using: 5 µL diluted (10×) product of preamplification, 0.5 µM of each selective primer (MspI-HpaII and EcoRI, Genomed) (Table 2), 1 U Taq DNA polymerase (Dream Taq, Thermo Scientific), 1× PCR buffer (Dream Taq, Thermo Scientific), and 200 µM of each dNTP in a final volume of 20 µL. The selective primers (MspI-HpaII and EcoRI) included two or three additional selective oligonucleotides (Table 2).

The selective amplification reaction consisted of two rounds with ‘touch-down’ in the first one. In the first round (12 cycles), the following profiles were used: denaturation at 94 °C for 30 s, primer annealing at 65 °C for 1 min reduced by 0.7 °C per cycle, primer extension at 72 °C for 1 min. In the second round (24 cycles), the reaction consisted of: denaturation at 94 °C for 30 s, primer annealing at 56 °C for 1 min and 72 °C for 1 min, with a final extension at 72 °C for 5 min. All amplification reactions were performed in a thermocycler (Biometra).

The products of selective PCR were separated using 6% denaturing polyacrylamide gel during electrophoresis according to descriptions by Stadnik et al. [15].

DNA fragments were detected by the silver staining method following Bassam and Gresshoff [116]. The DNA band patterns were confirmed by repeating the experiments twice.

MSAP techniques were analyzed according to Xu et al. [117]. A DNA methylation event was detected when bands, initially present in the gel from the reaction EcoRI + MspI (M), were absent from the reaction EcoR I + HpaII (H). In this case, with the “symmetric or fully methylation” defined, the internal cytosine of the 5′CCGG 3′ sequence was methylated (5′C^m^CGG 3′). Simultaneously, the presence of a band in H and absence in M indicated that the external cytosine of one DNA 5′CCGG 3′ sequence strand was methylated (5′^m^CCGG 3′). This was regarded as the “hemi-methylated state”.

The percentage methylation was calculated according to Xiangqiana et al. [118], as below:

Methylation (%) = (number of methylated bands)/total number of bands) × 100.

### 4.5. Statistical Analysis

The statistical analysis was performed using TIBCO Statistica 13.3.0 (TIBCO Software Inc., Palo Alto, CA, USA). In order to check the normality of the distribution at *p* = 0.05, the Shapiro–Wilk test was performed. The homogeneity of variance was checked. The repeated measures ANOVA test was then performed. In order to determine and verify the relationship, Tukey’s post hoc test was performed with a significance level of *p* ≤ 0.05. Error bars in the figures indicate the mean standard error (S.E.).

## 5. Conclusions

The aim of this study was to evaluate the effect of Si foliar application on the photosynthetic apparatus, gas exchange, stress factor content and the level of methylation of maize plants cultivated in varied soil salinity conditions. The conducted studies confirmed the positive effect of Si on the relative chlorophyll content, selected parameters of chlorophyll fluorescence and gas exchange of plants. Nevertheless, the effect of Si depended on the soil salinity (NaCl dose). At high soil salinity levels, a higher CCI content was found after the application of 0.2 and 0.3% Si. However, the parameters of chlorophyll fluorescence (PI, F_V_/F_0_, F_v_/F_m,_ and RC/ABS) were higher after spraying with Si at a dose of 0.3 and 0.4%, compared to lower doses (0.1 and 0.2% Si) and without Si application. Plant gas exchange parameters (Ci, P_N_, g_s_, E) were also higher after Si application, but in each Si dose (0.1–0.4%), the outcome depended on the soil salinity.

A higher content of chlorophyll *a, b* and *a + b* was found after the application of 0.3% Si at the highest soil salinity. The content of carotenoids was the highest after the application of Si at a dose of 0.2%. A lower content of free proline in the leaves, especially after the application of the highest dose of Si, was recorded in each salinity variant. It can be inferred that the application of Si reduces the negative effect of high soil salinity on maize plant growth.

The overall DNA methylation levels of maize varied among plants differentially treated. Decreasing methylation frequency was observed for maize plants under 100–300 mM NaCl salinity conditions, in combination with different doses of Si. Diminishing methylation frequency may indicate activation of the genes responsible for coping with stress conditions. It can be assumed that maize plants subjected to moderate salinity stress (100–300 mM) in combination with Si (0.1–0.4%) will improve their response to stress conditions in the next round of salinity or in the next generation. This could be due to the effect of methylation changes occurring during salinity stress conditions and the acquisition of epigenetic memory.

The obtained results of this research should be verified in the field, where various environmental factors can modify the reaction of plants to stress conditions and the results of Si foliar application.

## Figures and Tables

**Table 1:** Percentage scores of methylation events.

Variants		Type of Methylation		
NaCl	Si	Fully (Symmetric)	Hemi-Methylation	Total
0	0.0%	18.8	34.6	53.4
0	0.1%	17.5	37.5	55.0
0	0.2%	24.2	28.8	53.0
0	0.3%	18.1	39.6	57.7
0	0.4%	23.1	36.5	59.6
100	0.1%	15.6	26.2	41.8
100	0.2%	18.2	25.8	43.9
100	0.3%	17.1	28.9	46.1
100	0.4%	14.3	31.4	45.7
200	0.1%	23.3	26.7	50.0
200	0.2%	18.3	37.4	55.7
200	0.3%	15.4	37.6	53.0
200	0.4%	20.2	37.8	58.0
300	0.1%	16.0	34.4	50.4
300	0.2%	21.7	28.7	50.4
300	0.3%	17.7	35.5	53.2
300	0.4%	29.8	29.8	59.6
400	0.1%	28.1	35.1	63.2
400	0.2%	20.2	42.7	62.9
400	0.3%	22.7	39.4	62.1
400	0.4%	22.0	42.4	64.4

**Table 2:** Sequences of adapters and primers used for MSAP analysis.

MSAP Stage	Primer/Adapter	Sequence
Ligation	EcoRI-Adapter	5′CTCGTAGACTGCGTACC 3′ 3′CATCTGACGCATGGTTAA 5′
	MspI-HpaII-Adapter	5′CGACTCAGGACTCAT 3′ 3′TGAGTCCTGAGTAGCAG 5′
Preamplification	Pre-EcoRI	5′GACTGCGTACCAATTC 3′
	Pre-MspI-HpaII	5′GATGAGTCCTGAGTCGG 3′
Selective amplification	EcoRI-ACT	5′GACTGCGTACCAATTCACT 3′
	EcoRI-AC	5′GACTGCGTACCAATTCAC 3′
	EcoRI-AT	5′GACTGCGTACCAATTCAT 3′
	MspI/HpaII-ATG	5′GATGAGTCCTGAGTCGGATG 3′
	MspI/HpaII-CTA	5′GATGAGTCCTGAGTCGGCTA 3′
	MspI/HpaII-CT	5′GATGAGTCCTGAGTCGGCT 3′
	MspI/HpaII-GT	5′GATGAGTCCTGAGTCGGGT 3′

## Data Availability

The data presented in this study are available from the corresponding author upon reasonable request.
